# Novel Low-Cost Thermotherapy for Cutaneous Leishmaniasis in Peru

**DOI:** 10.1371/journal.pntd.0002196

**Published:** 2013-05-02

**Authors:** Braulio M. Valencia, David Miller, Richard S. Witzig, Andrea K. Boggild, Alejandro Llanos-Cuentas

**Affiliations:** 1 Instituto de Medicina Tropical “Alexander von Humboldt”, Universidad Peruana Cayetano Heredia, Lima, Peru; 2 Section of Infectious Diseases, Department of Medicine, Harbor-UCLA Medical Center, Los Angeles, California, United States of America; 3 Section of Infectious Diseases, Tulane University School of Medicine and East Jefferson General Hospital, New Orleans, Louisiana, United States of America; 4 Tropical Disease Unit, Division of Infectious Diseases, Department of Medicine, University Health Network and the University of Toronto, Toronto, Canada; 5 Hospital Nacional Cayetano Heredia, Lima, Peru; National Institute of Allergy and Infectious Diseases, United States of America

## Abstract

Thermotherapy is an accepted alternative therapy for new-world cutaneous leishmaniasis, but current heat-delivery modalities are too costly to be made widely available to endemic populations. We adapted a low-cost heat pack named the HECT-CL device that delivers safe, reliable, and renewable conduction heat. 25 patients with cutaneous leishmaniasis completed treatment with the device at an initial temperature of 52°C±2°C for 3 minutes to each lesion, repeated daily for 7 days, and were followed up for 6 months by direct observation. The overall definitive clinical cure rate was 60%. Concurrently, 13 patients meeting minimally significant exclusion criteria received identical compassionate use treatment with a cumulative definitive cure rate of 68.4%, 75% for those who had experienced CL relapse after prior antimonial treatment. Therapy was well tolerated. Reversible second-degree burns occurred in two patients and no bacterial super-infections were observed. HECT-CL is a promising treatment and deserves further study to verify its safety and efficacy as adjuvant and mono- therapy.

## Author Summary

American cutaneous leishmaniasis is an endemic parasitic disease in Peru, with more than a reported thousand cases per year associated with significant disabilities and economic impact. In comparison to old-world cutaneous Leishmaniasis where the infection generally heals on its own, American cutaneous Leishmaniasis is a chronic skin disease and its treatment can be expensive, difficult to administer, and associated with significant adverse events. We developed a heat treatment using a novel and low-technology device named Hand-held Exothermic Crystallization Thermotherapy for Cutaneous Leishmaniasis (HECT-CL). This device produces a stable thermal reaction ranging from 50–54°C which is considered therapeutic and has enormous benefits for its application in real world conditions, as it is extremely low cost, simple to use, and highly tolerable in patients receiving this treatment. Our pilot evaluation found cure rates close to those reported with the standard antimonial treatment and likely higher in cases of relapsing disease. Additionally, fewer severe adverse events were observed than reported with the use of other currently available heat therapies. Our objective is to show that HECT-CL therapy could be instituted in rural and real situations with limited health care infrastructure and with less cost and fewer side effects than standard antimonial treatment.

## Introduction

Cutaneous leishmaniasis (CL) is a sand fly transmitted protozoan disease on the WHO list of Neglected Tropical Diseases with an estimated incidence of l.5 million new cases yearly [1]. Peru hosts *L. (V) braziliensis and L. (V) guyanensis*, which are geographically distributed in the jungle, and *L. (V) peruviana*, which is distributed in the Andean highlands [2]. Administration of the first-line chemotherapy of pentavalent antimonials (Sb5+) is burdened by a high cost, a 20-day treatment course under expert supervision, frequent side effects [3]–[6], and rising clinical resistance [7]–[9]. Amphotericin B (AMB) is the only available second-line therapy in Peru, and is even more problematic with respect to cost, side effects, and treatment delivery [10], [11].

Berman and Sacks demonstrated several pathogenic *Leishmania* species to be thermosensitive from 37 to 39°C *in vitro* [12], [13]. Subsequently, thermotherapy (TT) has been evaluated in a variety of CL species and via a variety of heat-delivery modalities [14]–[17]. The ThermoMed™ device, which utilizes radio-frequency (RF) technology remains the most supported by randomized clinical trials and is WHO recommended as an alternative therapy for all American CL species [1]. In Peru, the high cost of this device, healthcare infrastructure limitations, and poverty in endemic areas has restricted its use to research and military settings [18]–[20].

We adapted a reliable, safe, and low-cost technology named the Hand-held Exothermic Crystallization Thermotherapy for Cutaneous Leishmaniasis (HECT-CL). The HECT-CL is a sodium acetate heat pad calibrated to produce 52±2°C for greater than 3 minutes; it costs less than 3 dollars, is simple to use, and is rechargeable by boiling for recurrent reuse. A supersaturated sodium acetate solution and flexible metal disc are contained inside a sealed plastic pouch. Flexing the disc provides a nanoscopic nidus that nucleates an exothermic liquid-to-solid phase change reaction releasing a reliable maximum temperature at 52±2°C. The liquid phase is restored when boiled. Heat packs operating with equivalent technology have been sold commercially as hand warmers and for treatment of athletic injuries [21], and sodium acetate is a food additive and is commonly infused intravenously with parental nutrition. In this study we evaluate the safety and efficacy of HECT-CL in in Peruvian patients with CL who had previous sodium stibogluconate (SSG) treatment and in those not previously treated with *L. (V) peruviana* infection

## Materials and Methods

### Participants/Study Location

The pilot study was performed at the Leishmaniasis Clinic, Institute of Tropical Medicine Alexander von Humboldt – Hospital Nacional Cayetano Heredia, in Lima, January through December 2011. Twenty-five subjects with parasitologically confirmed CL who were likely infected in Peru were included. Exclusion criteria were (1) age under 8 or older than 80 years old, (2) facial lesions located less than 2 centimeters from mucosal surfaces (such as the nose, mouth, eyes or ears), (3) maximum area of 15 cm^2^ (diameter greater than 4 centimeters), (4) more than 4 lesions, (5) *L. (V) braziliensis* or *L. (V) guyanensis* disease without prior systemic therapy, (6) concomitant mucosal leishmaniasis (ML), (7) severe or immunocompromising medical illness, (8) having received therapy for CL in the prior month, or (9) inability to commit to follow-up appointments for the proximal six months.

### Ethics Statement

The study received Institutional Review Board approval from both Tulane University School of Medicine and Hospital Nacional Cayetano Heredia. All adult participants provided written informed consent. Children less than 18 years of age were only included if written informed consent was provided by the participant's parent or guardian in addition to written agreement from the participant. This clinical trial was registered in Clinicaltrials.gov (NCT01277796).

### Baseline Evaluations

Subjects underwent a thorough history and physical examination with particular attention to exclude mucosal involvement. Lesions were measured using a digital caliper to record the largest extending diameter of and the corresponding perpendicular diameter. Standardized coded digital photographs were taken. Specimens for parasitologic testing were collected by scraping and non-invasive sampling, and diagnosis and speciation were confirmed by conventional PCR [22].

### Outcome Measures

The primary outcome measure was efficacy of HECT-CL according to prior treatment status and the causative species (*L.(V). braziliensis*, *L. (V). peruviana*, or *L. (V). guyanensis*). Treatment response (TR) was staged using the following clinical criteria:

M0: No improvement. Lesion remained active, having the same characteristics or becoming larger than prior to the start of treatment.

MI: Size of the lesion decreased 50% in comparison with the initial lesion, with fewer inflammatory signs and discrete re-epithelialization.

M2: Size of the lesion decreased between 50–90% in comparison with the initial lesion, and left few inflammatory signs.

M3: Size of the lesion decreased more than 90%, with re-epithelialization and very little inflammation.

M4: Complete re-epithelialization with a characteristic scar and no inflammation.

Standardized clinical evolution was evaluated based on the aforementioned TR staging as:

- Improvement: Stages M1–M3 before 3 months follow-up.
- Clinical Cure: Stage M4.
- Definitive Clinical Cure: Stage M4 at 6 months follow-up.
- Failure: Any one, or combination, of the following options:
  - Stage M0 at 1 month of follow-up after the treatment
  - Stage M0–M3 at 3 months of follow-up after the treatment
  - Regression of the clinical stage upon any follow-up evaluation

Secondary outcomes included pain, burn grade, and super-infection. Pain was evaluated by the Baker-Wong Likert emoticon-word rating scale [23]. Burn grade was noted as first-degree by the presence of erythema, or reversible (partial thickness) second-degree burn by painful reversible blistering. Super-infection was screened for by any persistent or progressive erythema or purulence.

### Study Intervention

**Figure 1:**
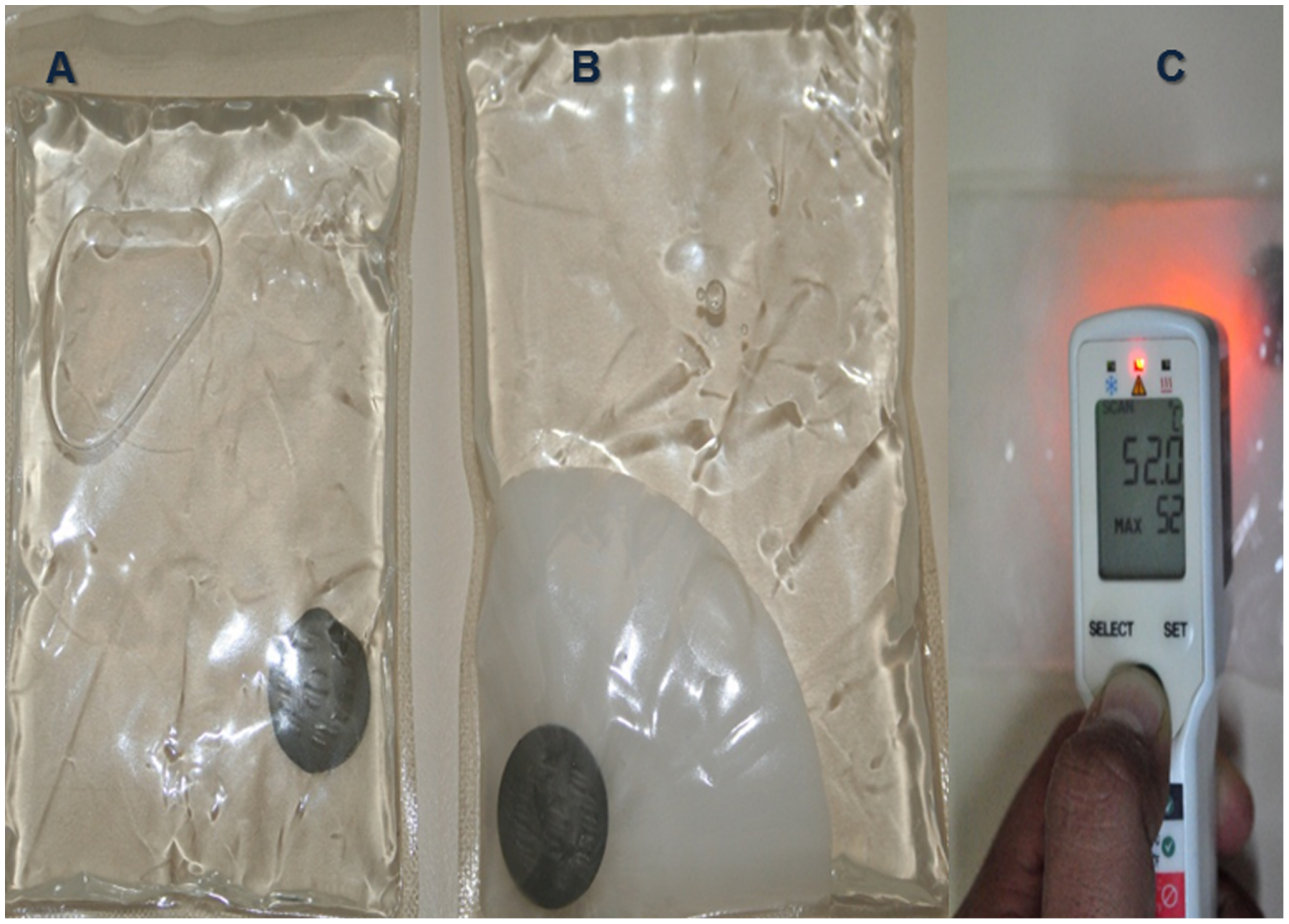
HECT-CL device activation and verification. (a) HECT-CL device before activation; (b) after activation, the supersaturated solution crystallizes; and (c) before application, temperature was measured using an infrared thermometer.

Each lesion was debrided and cleaned with sterile physiologic saline solution and the HECT-CL device activation temperature of 50–54°C was verified by an infrared thermometer (Figure 1, a–c). The HECT-CL device was applied only if temperature was in the range of 51–53°C given the ±1°C limitation in thermometer accuracy. The device was applied daily for 3 minutes in 1–3 fractions (according to individual pain tolerance) for 7 days; every application included at least 1 cm of “healthy skin” outside of the CL lesion. HECT-CL devices are malleable and the application area was delimited by careful hand application. Injectable lidocaine was available by request prior to direct application of HECT-CL. Local adverse events after HECT-CL were evaluated before and 30 minutes after heat therapy on days 1–7 and on scheduled clinical follow-up at 2 weeks, 1-, 2-, 3-, and 6 months. Study protocol allowed for HECT-CL interruption and initiation of a second-line therapy as soon as clinical suspicion of worsening was identified according to medical and ethical principles and oriented to reduce cosmetic implications especially in sensitive cosmetic lesions.

### Parasitological Confirmation and Strain Identification

CL was confirmed by direct amastigote identification on smear (Giemsa staining) and/or kDNA PCR. The principal causative species in Peru, *L. (V.) braziliensis*, *L. (V.) peruviana*, and *L. (V.) guyanensis* were differentiated by sequence targeted assays following the stepwise approach described by Veland, et al: a) Mannose phosphate isomerase gene (MPI) which distinguishes *L. (V.) peruviana* from *L. (V.) braziliensis* and *L. (V.) guyanensis*, b) The cysteine proteinase B (Cpb) gene which distinguishes between *L. (V.) braziliensis* and non-*L. (V.) braziliensis* species, c) Heat shock protein 70 (hsp70) which distinguishes between *L. (V.) guyanensis* and non-*L. (V.) guyanensis* species and d) an 870-bp fragment of *Leishmania* glycoprotein of 63 kDa (gp63) [24].

### Statistical Methods

Statistical analyses were performed using STATA software, version 10.0. Student's 2-tailed *t* test was used to compare the means of continuous variables (i.e., age, duration of disease, and number of lesions). Median values were compared using the Wilcoxon rank-sum test. Differences in clinical status were compared using the Fisher's exact test. Patients were analyzed as a group and then stratified according to prior treatment status. Differences were considered significant when *p* values were <0.05.

## Results

**Figure 2:**
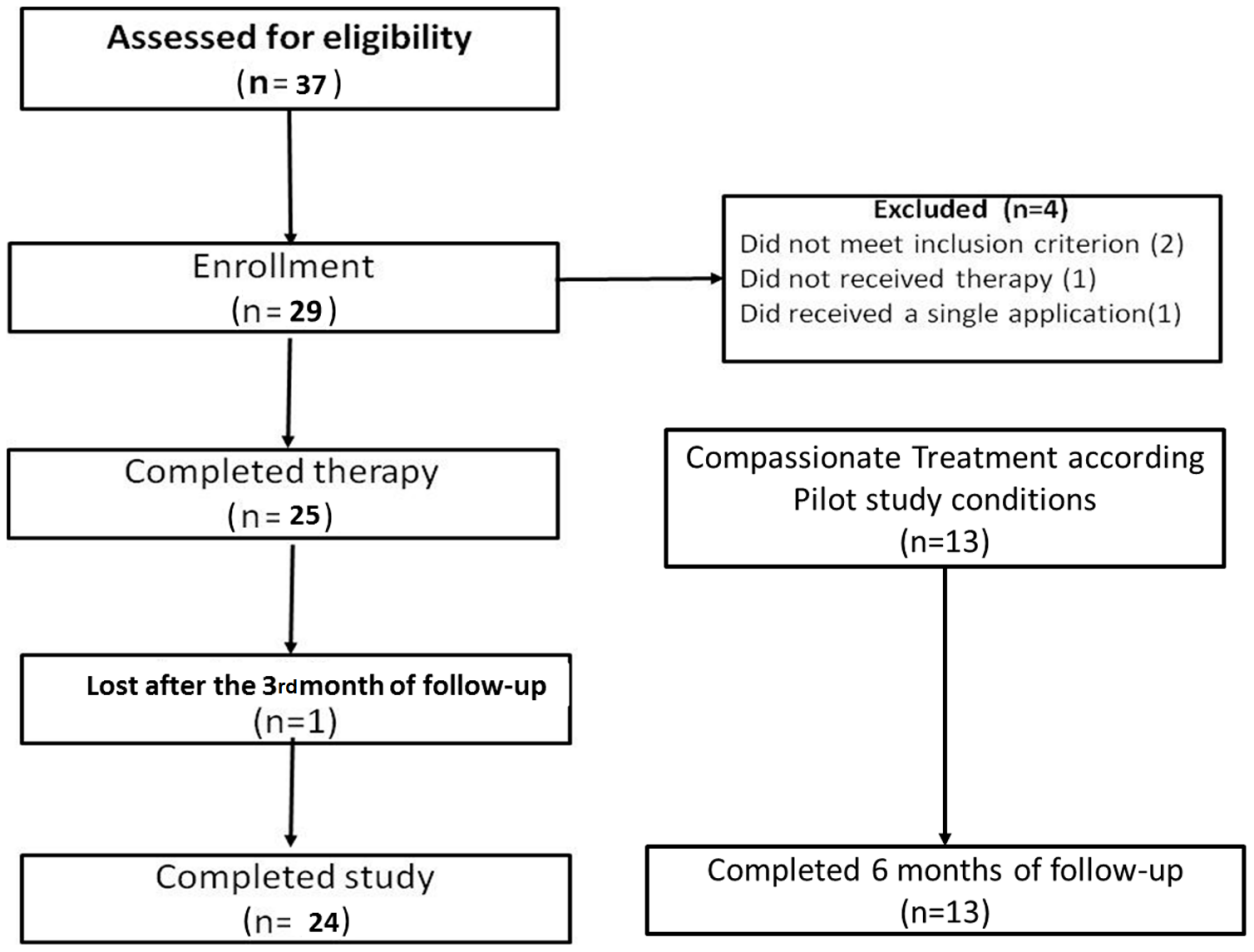
Study flow diagram.

Between January and June 2011, 37 patients were assessed for eligibility, 29 were enrolled and four were subsequently excluded: two patients were subsequently determined to have received prior therapy within 3 weeks of enrollment, and 2 other patients were lost to follow up secondary to social hardship. Twenty-five patients completed the study treatment: 16 males and 9 females. Adherence to follow-up was 100% through the 3rd month follow-up visit after; one patient did not follow up at 6 months (Figure 2).

Baseline demographic characteristics according to prior treatment history are shown in Table 1. By design, the naïve-to-treatment (NT) group included only *L. (V) peruviana* infection, whereas the prior-treatment (PT) group included infection by *L. (V) peruviana, L. (V) guyanensis and L. (V) braziliensis*. Prior-treatment patients were younger than those from the NT (p = 0.723). Patients with prior treatment had older lesions than those from the NT group (p = 0.041). The lesions in the PT and NT groups had a comparable size (p = 0.226). The predominant lesion was ulcerative independently of history of prior treatment (p = 0.117). Lesions were located more frequently on upper and lower limbs in both groups (p = 0.427).

**Table 1:** Baseline demographic characteristics.

Characteristic	n = 25	NT (n = 13)	PT (n = 12)	p value
Male sex (%)	16 (64)	6 (46.2)	10 (83.3)	.097
Median age in years (range)	25 (12–78)	25 (12–55)	25.5 (12–78)	.723
Occupational risk factor (%)				.216
- Agriculture	12 (48)	8 (61.5)	4 (33.3)	
- Child in school	6 (24)	3 (23.1)	3 (25)	
- Tourism	2 (8)	2 (15.4)	-	
- Oil activities	2 (8)	-	2 (16.7)	
- Other	3 (12)	-	3 (25)	
Andean Leishmaniasis (%)	17 (68)	13 (100)	4 (33.3)	.000
Median duration lesion in weeks, (range)	22 (6–141)	15 (6–103)	36 (11–141)	.041
Median number of lesions (range)£	1 (1–4)	2 (1–4)	1 (1–4)	.430
Ulcerative lesion (%)£	35 (81.4)	14 (73.7)	21 (87.5)	.117
Localization of lesion:£				.427
- Face	7 (16.3)	3 (15.8)	4 (16.7)	
- Upper limb	23 (53.5)	9 (47.4)	14 (58.3)	
- Lower limb	11 (25.6)	5 (26.3)	6 (25)	
- Abdomen	2 (4.6)	2 (10.5)	-	
Size of lesion in cm^2^ (range)£	2 (0.07–14.9)	2.63 (0.07–10.3)	0.94 (0.11–14.9)	.0226
Associated lymphadenopathy (%)£	8 (18.6)	5 (26.3)	3 (12.5)	0.432
Positive smear (%)	16 (64)	8 (61.5)	8 (66.7)	.16
Positive LST (%)§	14 (93.3)	7 (87.5)	7 (100)	1
Species identification				.006
- *L. (V). braziliensis*	6 (24)	-	6 (50)	
- *L. (V). peruviana*	16 (64)	12 (92.3)	4 (33.3)	
- *L. (V). guyanensis*	3 (12)	1 (7.7)	2 (16.7)	

£ Based on 43 lesions.

§ Only fifteen patients were tested with LST.

Abbreviations: AMB, amphotericin B; LST, leishmanin skin test; NT, naïve treatment; PT, prior treatment.

The temperature of the HECT-CL device just before and after application were similar between the NT and PT two groups (starting on average at 52°C and dropping to 48°C after heat delivery) (Table 2). Adults tolerated heat delivery well with a single 3-minute application whereas children typically required 2–3 fractions daily due to discomfort that varied with body location – the face lesions typically requiring more fractions than the limbs (Table 3). Pain of heat application as evaluated by the Baker-Wong scale was, at worst, rated moderate, and perceived to be more intense during the first application, waning on subsequent treatment days. No pain was reported 30 minutes and 24 hours after HECT-CL in any patient. Intervention-related pain and erythema resolved by 30 minutes after heat application and remained absent at 24 hours. Blistering occurred in two different patients identified 24 hours after heat delivery, both on the forearm, both of which healed completely without burn scar within 3 weeks. Of note, both cases of blistering resulted after heat application with initial heat pack temperature of 53°C. No patient developed irreversible burn or bacterial superinfection.

**Table 2:** Response to HECT-CL among groups based on prior history of antimonial treatment.

	n = 25	NT (n = 13)	PT (n = 12)	p value
a) **Temperatures**				
HECT-CL before application, (SD)	52.3 (0.7)	52.2 (0.7)	52.4 (0.7)	.367
Skin after application, (SD)	44.1 (1.2)	44.2 (0.8)	44 (1.5)	.703
b) **Time after the end of therapy, response**				
At the end of HECT-CL				.706
- Not improved	1 (4)	1 (7.7)	-	
- Improved	23 (92)	11 (84.6)	12 (100)	
- Cured	1 (4)	1 (7.7)	-	
At day 15				.21
- Not improved	2 (8)	2 (15.3)	-	
- Improved	13 (52)	4 (30.9)	9 (75)	
- Cured	10 (40)	7 (53.8)	3 (25)	
At month 1				.337
- Failure	3 (12)	1 (7.7)	2 (16.7)	
- Not improved	2 (8)	1 (7.7)	1 (8.3)	
- Improved	4 (16)	2 (15.4)	2 (16.7)	
- Cured	16 (64)	9 (69.2)	7 (58.3)	
At month 2				.305
- Failure	7 (28)	3 (23.1)	4 (33.3)	
- Not improved	2 (8)	1 (7.7)	1 (8.3)	
- Improved	1 (4)	1 (7.7)	-	
- Cured	15 (60)	8 (61.5)	7 (58.3)	
At month 3				.145
- Failure	8 (32)	4 (30.8)	4 (33.3)	
- Not improved	2 (8)	1 (7.7)	1 (8.3)	
- Improved	-	-	-	
- Cured	15 (60)	8 (61.5)	7 (58.3)	
At month 6				. 145
- Failure	9 (36)	5 (38.5)	4 (33.3)	
- Cured	15 (60)	8 (61.5)	7 (58.3)	
- Lost to follow-up	1 (4)		1 (8.4)	
**c) Cure rates by species**		**Definitive Cure**	**Treatment failure**	
*L. (V). braziliensis*	6	3	3	
*L. (V). peruviana*	16	9	7	
*L. (V). guyanensis*	3	3	-	

**Table 3:** Common adverse events by temperature during application.

	Day 1		Day 4		Day 7	
Adverse event	50–52°C	52–54°C	50–52°C	52–54°C	50–52°C	52–54°C
***Erythema, number %***	10 (100)	15 (100)	12 (100)	13 (100)	10 (100)	15 (100)
***Local edema, number (%)***	-	-	-	-	-	-
***Pain, number (%)***						
*- No pain*	1 (10)	2 (13)	1 (8.3)	1 (7.7)	1 (10)	1 (6.7)
*- Hurts a little bit*	6 (60)	5 (33.3)	9 (75)	9 (69.2)	9 (90)	12 (80)
*- Hurts a little more*	3 (30)	6 (40)	2 (16.7)	3 (23.1)	-	2 (13.3)
*- Hurts even more*	-	2 (13.3)	-	-	-	-
*- Hurts a whole lot*	-	-	-	-	-	-
*- Hurts worst*	-	-	-	-	-	-
***Blisters***	-	1 (6.7)	-	1 (7.7)	-	-
***Median age, (range)***	22.5 (12–52)	28 (12–78)	22.5 (12–52)	36 (12–78)	23.5 (12–55)	25 (12–78)
***Location (%)***						
*- Face*	3 (30)	3 (20)	4 (33.3)	2 (15.4)	4 (40)	2 (13.3)
*- Upper limb*	4 (40)	9 (60)	7 (58.3)	6 (46.1)	5 (50)	8 (53.3)
*- Lower limb*	2 (20)	3 (20)	1 (8.3)	4 (30.8)	1 (10)	4 (26.7)
*- Abdomen*	1 (10)	-	-	1 (7.7)	-	1 (6.7)

**Figure 3:**
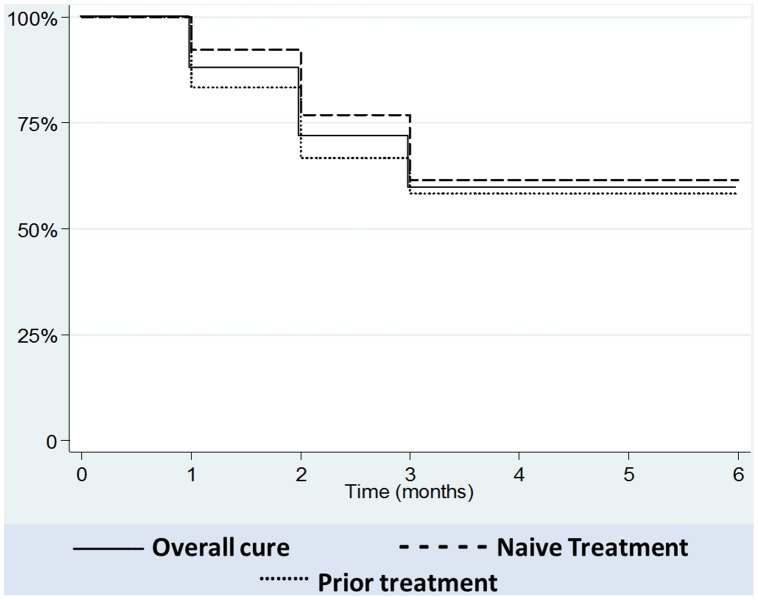
Kaplan-Meier curves.

**Figure 4:**
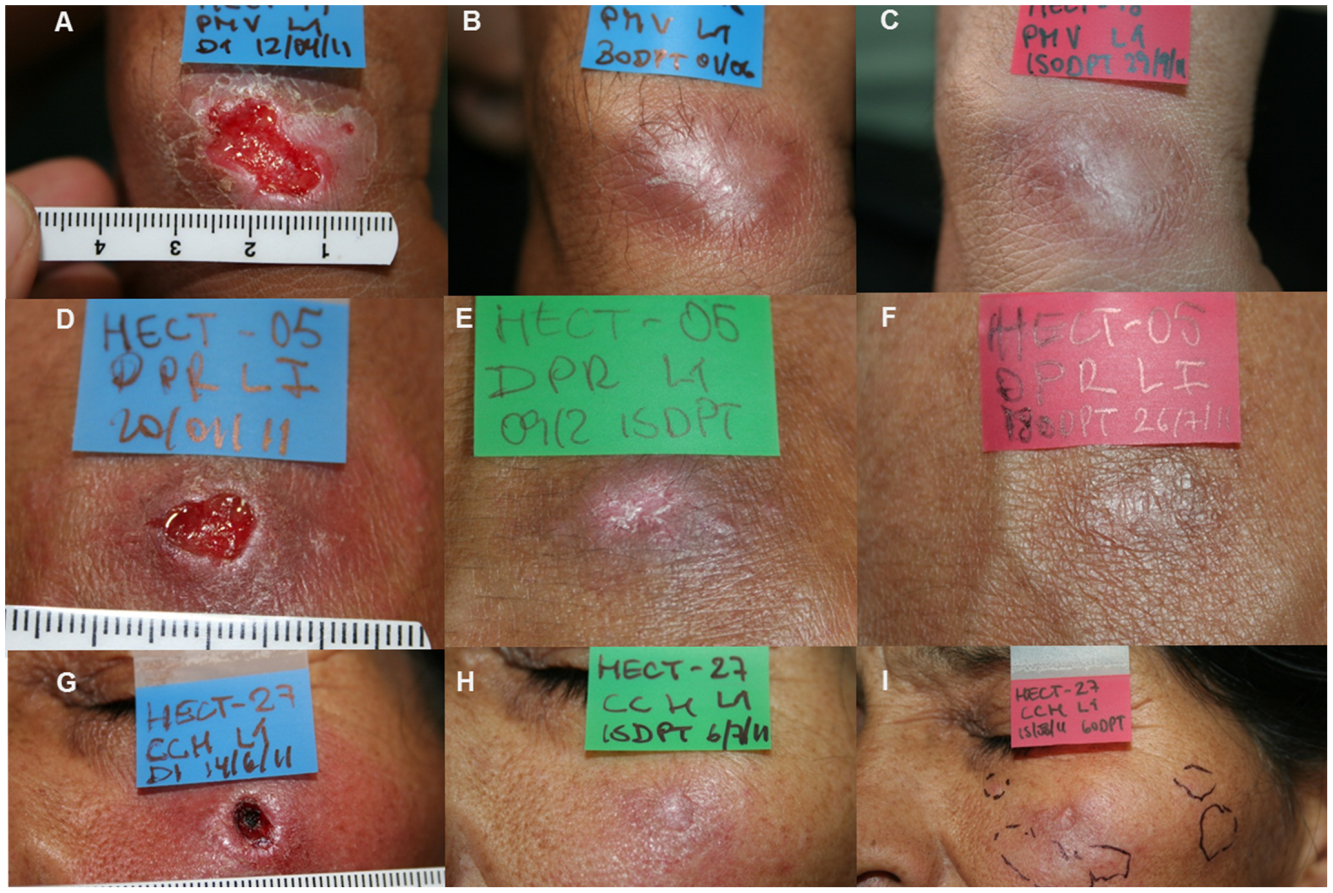
Response to HECT-CL therapy. a) Patient 19 (PT) at baseline; b) Patient 19 thirty days after HECT-CL with M4 clinical status; c) Patient 19 six months after HECT-CL with complete re-epithelialization; d)Patient 5 (PT) at baseline; e) Patient 5 fifteen days after HECT-CL with M4 clinical status; f) Patient 5 cured six months after HECT-CL; g) Patient 27 (NT) at baseline; h) Patient 27 fifteen days after HECT-CL with M4 status; i) Patient 27 two months after HECT-CL with complete re-epithelialization of lesion but 2 subcutaneous tracts.

Treatment response to HECT-CL is summarized in Table 2. The definitive clinical cure rate was 60% (15/25) without statistical difference between the NT (61.5%) and PT (58.3%) groups (Figure 3). Re-epithelialization and resolution of inflammation by one-month follow-up occurred in 64% of lesions with long term cosmetically favorable scaring (Figure 4a–f) for those achieving clinical cure. On day 7 of HECT-CL application 24 (96%) subjects had experienced improvement in stage, and one patient demonstrated clinical cure with complete re-epithelialization (M4). Clinical cure was subsequently observed in 10 subjects (40%) at 15 days after HECT-CL treatment initiation, 16 subjects (64%) at 1 month, and 15 subjects (60%) at 2 months; all 15 remained clinically cured at 3 and 6 months follow up.

Treatment failures were initially observed at 1 month when three patients (two with facial lesions) demonstrated re-ulceration accompanied by erythema and infiltration (M0). Two of these had a full resolution without cosmetic implications after standard antimonial therapy and one was unable to receive Amphotericin B and was lost to follow-up. At 3 months two others were classified as treatment failures due to their M2–M3 stage. Five of the 10 patients (two with facial lesions) who failed HECT-CL had a paradoxical behavior characterized by appearance of satellite subcutaneous nodules despite complete resolution of the treated ulcer (Figure 4g–i).

Clinical cure rate by species (Table 2) was 56.2% in those with *L. (V) peruviana* (9/16 patients), 50% in those with *L. (V) braziliensis* (3/6 patients), and 100% in those with *L. (V). guyanensis* (3/3). No patient with *L. (V) braziliensis* showed evidence of mucosal involvement on follow-up.

### Post-Hoc Analysis

Parallel to the 25 patients enrolled in this Pilot study, 13 additional patients were treated with HECT-CL on compassionate grounds because they were deemed ineligible to receive Sb5+ or AMB due to one or more of the following: age less than 8 years old, a social or financial conflict for a 20-day treatment course, a medical contra-indication, and/or previous failed treatment prior to HECT-CL therapy. Each patient or his or her legal representative signed an informed consent for publication of clinical information, which was approved by the UPCH Institutional Review Board. The 13 additional patients were largely female (61.5%) with a median age of 7 years (Range: 3–52) who acquired the infection predominantly in highland regions (76.9%). All patients had *L. (V) peruviana* as the causative parasite (eleven of whom had received prior Sb5+), with a median duration of disease of 26 weeks (range 8–106 weeks). The previously treated lesions generally had relapsed as nodules over the inactive post-treatment scar, later progressing with erythema and induration with a striking clinical similarity to Leishmaniasis Recidivans Cutis (LRC) described in old world CL [25]. At presentation these lesions tended to be multiple, nodular, and ulcerative: 12 of 13 patients had more than two lesions. At the end of HECT-CL therapy, day 7, ten of 12 subjects had stage improvement and one patient demonstrated complete re-epithelialization (M4). Clinical cure (M4) was subsequently observed in 6 of 13 patients at fifteen days after HECT-CL therapy end, 12 of 13 patients at 1 month, and all 13 patients at 2 months. However, at 3 months follow up, 1 patient experienced re-ulceration, and another patient the appearance of satellite subcutaneous nodules despite complete re-epithelialization of the treated ulcer. At 6 months after treatment, 11 of 13 patients experienced definitive clinical cure (84%).

Post-hoc analysis including the 13 patients treated compassionately and the 25 pilot study subjects, demonstrates a definitive clinical cure rate of 68.4% (26/38), 60% (9/15) in the NT group and 73.9% in the PT group.

## Discussion

This study demonstrates that HECT-CL is safe and is promising to be efficacious. While the natural evolution of untreated New World CL is still poorly described, the rapid improvement observed with HECT-CL is striking. The definitive clinical cure rate reported here of 60–68.4% rivals the efficacy estimates of pentavalent antimonials for treatment CL in South America of 76.5% [26], as well as broad variable efficacy of standard Radiofrequency thermotherapy (RF-TT) ranging from 38 to 90% with a variety of species [18]–[20], [27], [28]. The study also found HECT-CL to be associated with clinical cure of the three most common species in Peru, with the caveat that there may be regional variation in virulence and heat-susceptibility within species also endemic in other countries [29], [30]. Clinical cure rates of reactivated lesions after Sb5+ treatment have been reported to be even lower than in treatment naïve patients [31], suggesting that HECT-CL may be preferred in this setting.

The HECT-CL safety profile was notable for a cumulative reversible 2^nd^ degree burn rate of 0.8%, with 2 events out of 266 applications (with fractionated heat deliveries grouped as a single application). In comparison, standard RF-TT can be complicated by up to a 93% 2^nd^ degree burn rate and 19% wound infection rate per treatment [18]. While RF-TT heats dermal tissue directly, the HECT-CL conduction modality may enjoy a superior safety profile secondary to physiologic dermal-protective thermoregulation via vasodilatation and convection heat transfer. Of note, both episodes of HECT-CL reversible 2^nd^ burn were observed after treatment initiation at 53°C±1°C, suggesting to limit initial treatment temperatures to 52±1°C in future investigations. While the RF-TT standard dosing delivers 50°C over 30 seconds accurately and precisely, optimal heat delivery has not been assessed by phase 2 trial. HECT-CL devices increased skin temperature in a fixed range of 41–45°C that was unquestionably therapeutic and considerably better tolerated than RF-TT. In the combined group reported here, 37 of 38 patients demonstrated initial improvement after HECT-CL application with an excellent safety profile, suggesting a role for elongated or recurrent treatment courses in the unsatisfactory lesion response. The reason why our pilot study used an extended therapy (7 days) intervention contrasting with a single application of conventional RF-TT is due to concerns about the efficacy of a single application with HECT-CL, which was not tested before as a thermotherapy device for treatment of CL. Compared with the normal RF-TT device, there was no medical evidence to support that a single application of HECT-CL is similarly effective. For this reason it is necessary to design future controlled clinical trials considering modifications of current thermotherapy procedures to identify if prolongation or reduction of applications offers the best therapeutic outcomes. Future trials should be implemented by immunological studies to support or refute the systemic immune response hypothesis after local heat treatment and identified molecular and cellular phenomena during heat therapy.

The observation of some recently infected NT patients developing satellite subcutaneous lesions despite complete resolution of the index lesion (Fig. 4g–i) could indicate these resulted from a high parasite load disease. This phenomenon was previously reported by Unger et. al in patients with young lesions treated with Sb5+ in whom failure rate was comparatively higher than in patients with well-established ulcerative lesions [32]. This hypothesis is supported by our post-hoc analysis findings in which subjects were primarily antimonial experienced with lesions that were focal, that likely contain a low parasite load, and demonstrate higher clinical cure rate with HECT-CL (84%) than the pilot population. Still there is limited insight into the TT physical and biological mechanisms of action beyond the general concepts of heat-dependent parasite destruction augmented by local and systemic immunity [14]. Our findings highlight the importance of further study to better characterize species-specific amastigote heat-tolerance, parasite load and mechanism of extension, as well as host immunologic factors. In the meantime, our findings suggest the lesions most effectively treated with HECT-CL may be those with focal relapse after treatment, or older lesions (>2 months) with a lower parasite load.

There is reasonable concern that local therapy may not protect against mucocutaneous progression in those patients infected with *L. (V). braziliensis*. However, neither Sb5+ or other systemic treatments have been strongly demonstrated to protect against ML, and there is evidence that heat therapy may induce a systemic response [1]. All of our subjects infected with *L. (V). braziliensis* or *L. (V). guyanensis* had received prior standard Sb5+ treatment, and until ML protection is better characterized, caution should be exercised with rural field use of TT monotherapy in ML regions. In the meantime, TT remains a promising candidate for rural field use in Peruvian regions devoid of ML, and in the tertiary care centers to improve efficacy, safety, and cost of the current standard therapy. Our results suggest that *L. (V). braziliensis* can be refractory to HECT-CL as used in our protocol. However, our *L. (V). braziliensis* patients were not representative of typical *L.(V). braziliensis* infections since all were included after receiving prior unsuccessful standard pentavalent antimonial treatment. Theoretically *L.(V). braziliensis* might be more refractory to HECT-CL, as in the case of other therapies [29], but due to the absence of molecular or immunological evidence supporting lower thermosensitivity in *L.(V) braziliensis* strains, we are unable to speculate about differences in treatment outcome according to the infecting strain.

The potential benefit of a safe, low-cost, easy to use, and efficacious therapy is particularly relevant in low resource countries like Peru where standard Sb5+ or second-line non-liposomal AMB treatment course requires 20-days of directly observed treatment in a tertiary care setting. Such a commitment is often either prohibitive or financially catastrophic for low-income rural populations. Therefore, carefully designed exothermic crustallization conduction heat therapy should be explored as mono- or adjuvant therapy to make treatment more widely available in endemic regions, and to potentially shorten the duration of directly observed toxic and/or expensive therapy.
